# Middle Frontal Horizontal Partial Laryngectomy (MFHPL): A Treatment for Stage T1b Squamous Cell Carcinoma of the Glottic Larynx Involving Anterior Vocal Commissure

**DOI:** 10.1371/journal.pone.0052723

**Published:** 2013-01-09

**Authors:** Wen-bin Lei, Ai-yun Jiang, Li-ping Chai, Xiao-lin Zhu, Zhang-feng Wang, Yi-hui Wen, Zhen-zhong Su, Wei-ping Wen

**Affiliations:** Otorhinolaryngology Hospital, Otorhinolaryngology Institute, The First Affiliated Hospital, Sun Yat-sen University, National Key Department of Otorhinolaryngology of People's Republic of China, Guangzhou, People's Republic of China; University of Nebraska Medical Center, United States of America

## Abstract

**Objective:**

The therapeutic effect of middle frontal horizontal partial laryngectomy (MFHPL) in treating stage T1b squamous cell carcinoma of the glottic larynx involving anterior vocal commissure (AVC) was compared with that of the anterior frontolateral vertical partial laryngectomy (AFVPL). The feasibility and practical significance of MFHPL in clinical application was discussed in the present study.

**Methods:**

From January 1996 to January 2010, a total of 65 patients diagnosed with stage T1bN0M0 glottic laryngeal cancer were treated with MFHPL or AFVPL. The postoperative complications, glottic reconstruction, recurrence rate, voice quality and survival rates were evaluated and compared between two treatments.

**Results:**

AFVPL and MFHPL were performed in 34 and 31 patients, respectively. Flexible fiberoptic laryngoscopy revealed that in the MFHPL-treated patients the reconstructed glottis was spacious and symmetric. In contrast, AFVPL treatment resulted in irregular glottic area with poor symmetry and tubular glottis. The incidence of postoperative laryngeal stenosis significantly differed between the MFHPL- and AFVPL-treated groups (P = 0.025). No significant difference was detected in the 3- and 5-year overall- or tumor-free survival rates between two treatments. The Voice Handicap Index (VHI) and maximum phonation time (MPT) after surgery were 51.0±12.99 and 12.42±3.44 sec in the AFVPL-treated group; while in the MFHPL-treated patients they were 31.81±7.48 and 7.65±1.98 sec, respectively. Both differences in VHI (P = 0.012) and MPT (P = 0.024) were significant between two treatments.

**Conclusions:**

MFHPL was comparable to AFVPL with respect to postoperative complications, recurrence rate and survival rates, but possessed advantages over AFVPL in terms of the incidence of laryngeal stenosis and voice quality. Our study indicated that MFHPL has a potential value in clinical practice of treating stage T1b squamous cell carcinoma of the glottic larynx involving AVC.

## Introduction

Although stage T1b squamous cell carcinoma of the glottic larynx involving anterior vocal commissure (AVC) is an early-stage cancer, the clinical efficiency of current treatments for it is in general not satisfactory [1]–[4]. The commonly used clinical treatments include radiotherapy, transoral CO_2_ laser surgery (TLS) and open surgery. Compared to the other two treatments, radiotherapy preserves the highest voice quality in patients but is associated with more side effects and relatively high recurrence rate. It is believed that the poor prognosis of radiotherapy is partially attributed to the AVC involvement of tumors, and close follow-up is required to enable a rescue surgery on the recurrent tumor in time [3], [4], [5], [6]. There are controversial opinions regarding the efficiency of TLS on treating this type of carcinoma with AVC involvement. Given the difficulties in AVC exposure and deficiency of CO_2_ laser in cutting cartilage, TLS is also associated with a relatively high recurrence rate [2]–[4], [7], [8]. Types of open surgery include laryngofissure, window partial laryngectomy, AFVPL, supracricoid partial laryngectomy –cricohyoidoepiglottopexy (SCPL-CHEP), etc. However, postoperative issues such as development of glottis granuloma, laryngeal scar stenosis, poor phonation and recurrence have been long-term concerns in clinical work [1]–[4], [9].

Since 1996, 65 patients with stage T1b squamous cell carcinoma of the glottic larynx involving AVC received either MFHPL or conventional AFVPL procedure in our institute. We found that MFHPL had significant advantages over AFVPL in that in addition to complete removal of the tumor, MFHPL preserved an intact laryngeal framework, decreased the laryngeal wound, reduced the occurrence of laryngeal stenosis and improved the voice quality post surgery. The present study reports the comparison of clinical efficiency between these two procedures.

## Materials and Methods

### 1 (O)Ethic statement

The study was approved by the Education and Research Committee and the Ethics Committee of Sun Yat-sen University (approval # SYU0089301). Written informed consent was obtained from each patient, who granted us permission to use the data obtained in subsequent studies.

### 2 (I) Subject selection

Patients with stage T1bN0M0 early glottic carcinoma and anterior commissure involvement were investigated in our studies. All diagnoses were confirmed as squamous carcinoma by biopsies. Patients with TNM stages other than T1bN0M0 were excluded.

### 3 (II) Clinical data

65 cases of T1bN0M0 glottic carcinoma involving AVC (UICC-2002 TNM classification) were admitted into the department of Otolaryngology, the First Affiliated Hospital of Sun Yat-sen University from January 1996 to January 2011. Patients were treated surgically by either MFHPL or AFVPL procedure. 34 patients (31 males and 3 females) received AFVPL procedure, at age of 43–80 (median: 62); 31 patients (28 males and 3 females) received MFHPL procedure, at age of 45–84 (median: 63). All cases were confirmed by biopsies as squamous carcinoma. Tumor location, invasion, and lymph node involvement were evaluated before surgery by contrast-enhanced spiral CT and electronic laryngoscopy. None of the patients received pre- or post-operative chemoradiotherapy.

### 4 (III) Surgical procedures

Two surgical procedures of partial larygectomy were used in our studies for stage T1bN0M0 glottic squamous carcinoma.

#### (1) Middle Frontal Horizontal Partial Laryngectomy (MFHPL)

**Figure 1:**
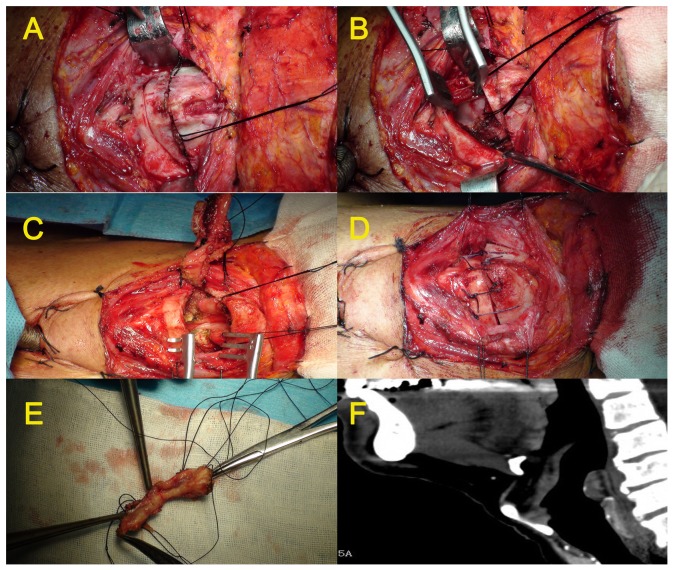
Diagram of MFHPL procedure. **A:** The thyroid cartilage was incised horizontally; **B:** Larynx was entered through the supra- or sub-glottis; **C:** Tissue defect after removal of the middle part of larynx together with tumor under direct vision; **D:** The larynx was tied and sutured; **E:** Resected tissue.; **F:** The anatomical relationship between the AVC and cartilage indicated by spiral CT.

**Figure 2:**
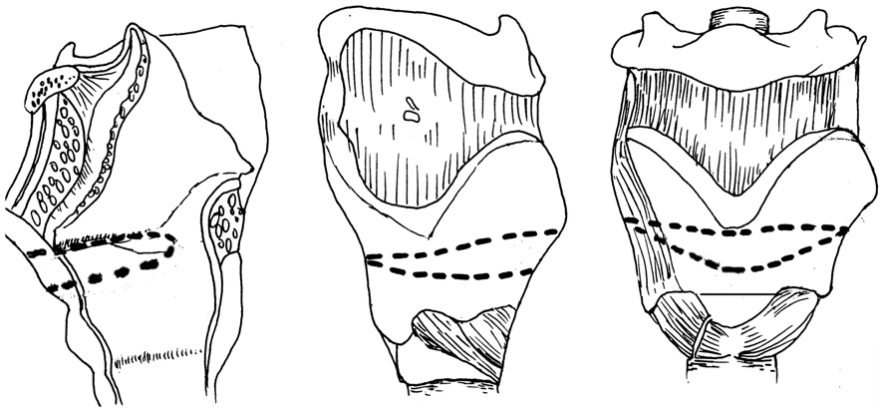
Schematic diagram of MFHPL. **A:** Dotted line indicates the resection area within the larynx during MFHPL; **B, C:** Dotted line indicates the resection area of thyroid cartilage during MFHPL.

A tracheostomy was routinely performed. A horizontal incision was made along the inferior aspect of the cricoid cartilage between the anterior margins of the bilateral sternocleidomastoid (SCM) muscles. Superior flap of the platysma was elevated superiorly and the underlying strap muscles were exposed. The bilateral sternohyoid muscles and thyroid cartilage perichondrium were separated inferiorly till the posterior border of the cartilage. A window of cartilage was made by cuts from upper 1/3 (about 0.5 cm to the superior aspect of the cartilage) and lower 1/3 (about 0.5 cm to the inferior aspect of the cartilage) in the midline to the middle of the posterior aspects of the cartilage (Figure 1A). Larynx was then entered by transecting the intralaryngeal mucosa/muscles through one of the cartilage incisions that was farther from the AVC (Figure 1B), based on the anatomical relationship between the AVC and cartilage indicated by CT (Figure 1F). The tumor was then resected under direct vision with tumor free margins of over 5 mm. The resection area was circumscribed by an upper bound of the free edges of the bilateral vestibular folds, a lower bound of more than 5 mm below the glottis, a posterior bound of the vocal process of arytenoid cartilage, and an anterior bound of the thyroid cartilage, to ensure the removal of whole tumor together with the intralarygeal tissues (Figures 1C, 2). Tumor free margins were biopsied at multiple foci over 5 mm from the tumors in all cases. Reconstruction: The glottis was reconstructed using the vestibular folds. To ensure the margins free of tumor, the anterior commissure of vestibular folds was removed when this region was adjacent to the tumors. In such cases, the anterior residues of the vestibular folds were sewed with the preserved anterior aspect of the thyroid cartilage to ensure the glottis reconstruction. To prevent postoperative adhesions, at least one side of the anterior commissure mucosa should be kept intact. With 3-0 Vicryl suture materials, stitches entered at the upper cartilage lamina and came out at the lower vestibular mucosa, then entered at the lower aspect of thyroid cartilage lamina and came out at the subglottic mucosa. These four stitches were aligned and tied to close the larynx, and the thyroid cartilage was then covered with the perichondrium of the sternohyoid cartilage (Figure 1D).

#### (2) Anterior Frontal Vertical Partial Laryngectomy (AFVPL)

**Figure 3:**
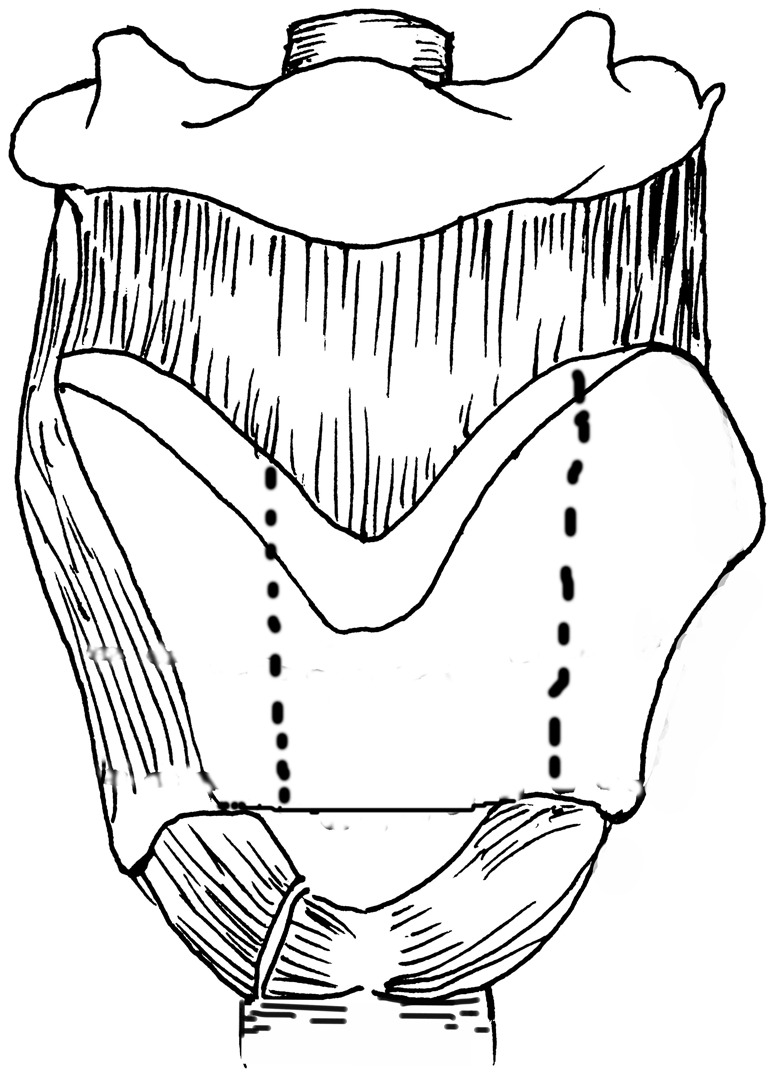
Schematic diagram of AFVPL. Dotted line indicates the resection area of thyroid cartilage during AFVPL.

The AFVPL procedure was conducted as previously described [10]. The vertical outline was made along the anterior aspect of the thyroid lamina with 3–5 mm deviation towards the less involved side, and approximately 8–12 mm deviation towards the more severely involved side(Figure 3). A vertical incision was made with a saw on bilateral thyroid laminas. The laryngeal cavity was visualized and approached through the less involved side. The true and false cords interior to the frontolateral thyroid cartilage were resected along with subglottic soft tissues, with tumor margins of over 5 mm. Depending on the extent of the laryngeal defects, the laryngeal cavity was closed directly or reconstructed using sternohyoid myofascia (single or double pedicles), or sternohyoid perichondrium.

### 5 (IV) Postoperative follow-up and evaluation

#### (1) General postoperative observational evaluation

The patients were observed for extubation, wound healing, and complications such as laryngeal stenosis, subcutaneous emphysema, pharyngeal/laryngeal fistula, aspiration and pneumonia. The patients were followed up for tumor recurrence and metastasis.

#### (2) The Voice Handicap Index (VHI) questionnaire

All patients in both surgical groups were evaluated 6 months post surgery for their voice by using the Chinese version of a VHI questionnaire proposed previously by Jacobson, et al [11]. This VHI questionnaire evaluates the impact of voice dysfunction on patients' quality of life in three aspects: Functional (F), Physical (P), and Emotional (E), with an overall evaluation as Global (G). A higher score in one aspect indicates that the patients are more negatively affected in that specific aspect. The magnitude of global score reflects the subjectiveness of patients in grading the impact of their voice dysfunction. The higher the G score is, the more subjective the patients are [11], [12].

#### (3) Vocal cord evaluation by electronic laryngoscopy

The laryngoscopy examination was performed to evaluate vocal cord activity and closure, as well as the size and symmetry of the glottis 6 months post operation. Potential complications such as granuloma, laryngeal web and stenosis were also examined in this procedure.

#### (4) Maximum phonation time (MPT)

MPT was evaluated in all patients 6 months post operation. Patients were asked to take one deep breath and then pronounce the vowel / a: / as long as possible at a comfortable pitch and loudness. The final MPT was obtained based on the best performance of three attempts (Normal values: >20 seconds for males; >15 seconds for females).

#### (5) Statistical analyses

t test and χ2 test were performed using SPSS 13 software and the survival was analyzed by Kaplan Meier analysis using SPSS 13 software.

## Results

From January 1996 to January 2011, a total of 65 patients diagnosed with stage T1b squamous cell carcinoma of the glottic larynx involving AVC were enrolled into the present study. These patients were assigned to either MFHPL or AFVPL procedure. The age, sex and pathological differentiation of tumors were comparable between these two groups, verifying the feasibility of subsequent comparisons on postoperative complications, efficiency of procedure and survival of patients (Table 1). All cases were confirmed as squamous cell carcinoma by postoperative pathological biopsies. Two cases from each group were found false negative at the tumor free-margin and radiation therapy (60GY) was applied after surgery. Two cases from each group were found to have mild thyroid cartilage involvement at anterior commissure and were pathologically re-graded as T3N0M0. No specific treatment was performed in these patients.

**Table 1:** Clinical parameters of patients.

Parameters	Procedure		
	AFVPL	MFHPL	P value
**Age (years)**			0.802
≤60	15	12	
>60	19	19	
**Sex**			1.000
Male	31	28	
Female	3	3	
**Differentiation**			0.976
G_1_	16	15	
G_2_	14	12	
G_3_	4	4	

### 1. Postoperative complications and recurrence

In the MFHPL group, postoperative complications included subcutaneous emphysema (n = 3), aspiration (n = 4) and pneumonia (n = 2); tumor recurrence occurred in four patients. Complications in the AFVPL group included subcutaneous emphysema (n = 5), aspiration (n = 5), pneumonia (n = 1) and laryngeal fistula (n = 2); tumor recurrence occurred in five patients. In the MFHPL group, extubations were successfully executed within one month after surgery without laryngeal stenosis observed. In contrast, extubations were not smoothly executed in the AFVPL group in which laryngeal stenosis was observed in six patients. The difference in the incidence of laryngeal stenosis between two procedures was statistically significant (0% versus 17.6%, P = 0.025). No significant difference was detected in the incidence of other complications and recurrence rate between two groups.

### 2. Voice quality assessment

In the MFHPL group, patients initially exhibited a low voice with impaired quality 1–2 months post surgery, which was significantly improved 3–4 months after the surgery. 6 months later, the patients were able to pronounce clearly with an average MPT of 12.42±3.44 s. In contrast, patients received AFVPL procedure encountered persistent difficulty in speech and exhibited a rough and low-pitched voice. The 6-month postoperative MPT value in AFVPL group (7.65±1.98 s) was significantly shorter than that in the MFHPL group (P = 0.024). VHI assessment was performed half year after the surgery and the results were summarized in Table 2. As shown, MFHPL procedure resulted in VHI scores that were significantly different from those resulted from the AFVPL procedure at aspect of functional (F), physical (P), emotional (E) and global (G) assessment (P<0.05).

**Table 2:** Comparison of VHI scores between MFHPL and AFVPL groups.

	Functional (F)	Physiological (P)	Emotional (E)	Global (G)
A	11.77±3.77	12.97±3.90	7.06±2.16	31.81±7.48
B	18.44±4.43	20.21±6.89	12.35±4.86	51.00±12.99

A: MFHPL; B: AFVPL. P value of t test all <0.05.

### 3. Electronic laryngoscopy analysis

**Figure 4:**
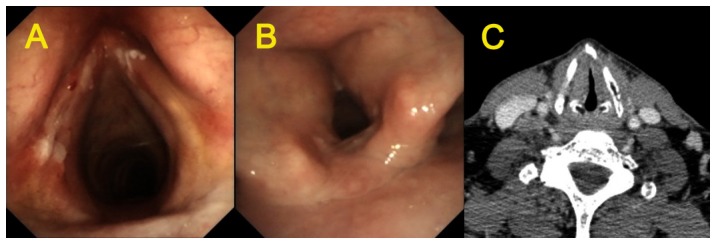
Pre- and post-surgery glottis of patients receiving MFHP. **A:** The preoperative glottis under laryngoscopy; **B:** The postoperative glottis under laryngoscopy 6 months after procedure. **C:** The preoperative glottis under contrast-enhanced spiral CT.

Electric laryngoscopy was performed to examine the glottis reconstruction. It was revealed that in the MFHPL group, the reconstructed glottis was spacious and symmetric, the arytenoid cartilage moved smoothly and the glottis was closed almost normally during phonation (Figure 4). In contrast, AFVPL treatment resulted in irregular glottic area with poor symmetry and tubular glottis with fair to poor closure during phonation. Three patients in each group developed glottis granuloma, which disappeared spontaneously 3 months after surgery. One patient in MFHPL group showed laryngeal webs at the AVC region, which might be attributed to the postoperative adhersions at the reconstructed AVC. The breathing was not affected in this patient. Five patients in the AFVPL group were found to form laryngeal webs adhered to the reconstructed AVC. The incidence of laryngeal webs was not significantly different between these two groups (P = 0.2).

### 4. Postoperative survival rates

In the MFHPL group, the follow-up time ranged from 15 to 120 months. A total of 20 patients were followed up for more than 3 years and 10 patients for more than 5 years with one patient lost. Tumor local recurrence occurred in four patients (13.3%) with one of them accompanied with neck lymph node metastasis. Four patients died from cardiovascular accident (n = 1), cerebral vascular accident (n = 1) or tumor recurrence (n = 2). The 3- and 5-year overall survival rates were 96.4% and 86.0%, respectively. The 3- and 5-year disease-free survival rates were 96.4% and 78.1%, respectively.

**Figure 5:**
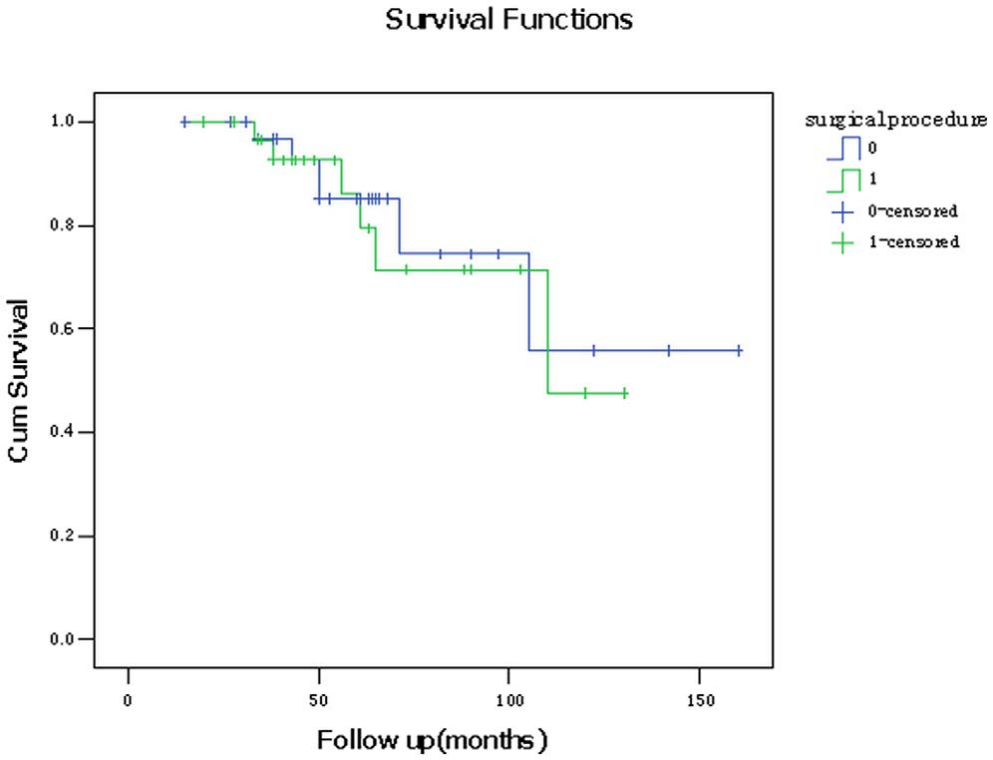
Kaplan-Meier curve for overall survival of 57 patients received either MFHPL or AFVPL procedure. 0: AFVPL group; 1: MFHPL group.

**Figure 6:**
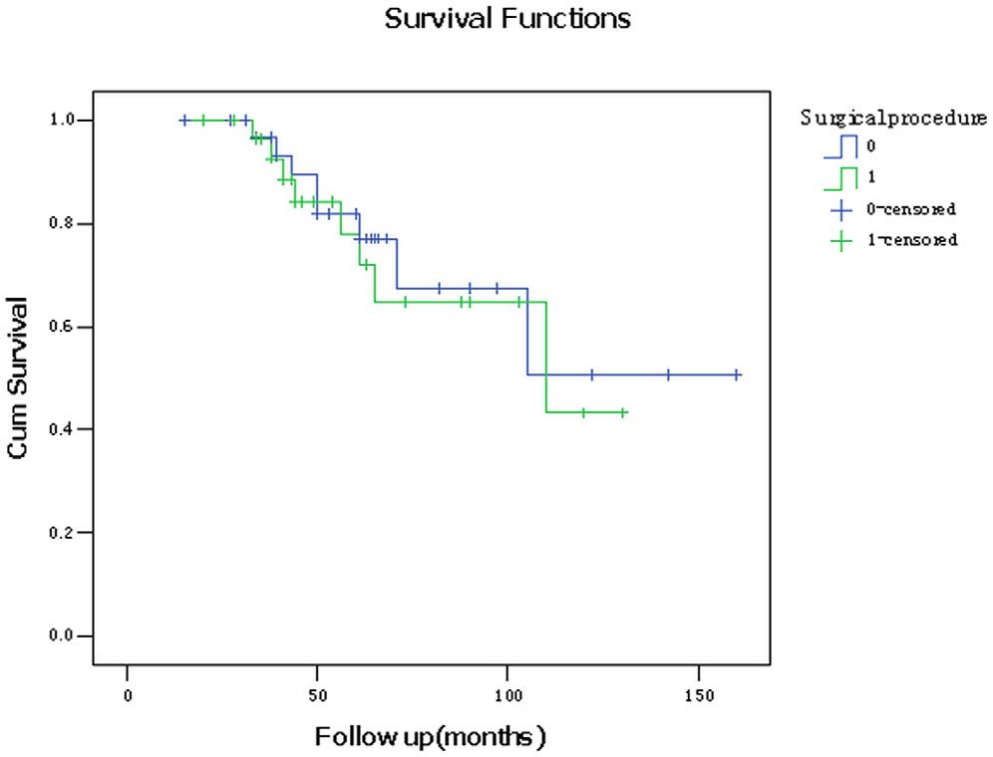
Kaplan-Meier curve for tumor-free survival of 57 patients received either MFHPL or AFVPL procedure. 0: AFVPL group; 1: MFHPL group.

In the AFVPL group, all the patients were followed up for 20–130 months. A total of 28 patients were followed up for more than 3 years with 2 patients lost. Tumor local recurrence occurred in 4 patients (15%) with two of them accompanied with neck lymph node metastasis. Four patients were died from chronic obstructive pulmonary disease (COPD, n = 1), tumor recurrence (n = 1) or unknown reasons (n = 1). The 3- and 5-year overall survival rates were 96.7% and 85.1%, respectively. The 3- and 5-year disease-free survival rates were 96.7% and 81.9%, respectively (Figures 5 and 6). The between-group difference in survival rates was tested by Kaplan-Meier analysis. No significant difference was detected in either the overall survival rates (Log Rank = 0.118, P = 0.732) or disease-free survival rates (Log Rank = 0.148, P = 0.701) between these two groups. Lost and died patients were considered censored cases in the survival analysis.

## Discussion

Glottic laryngeal tumors involving AVC have relatively high recurrence rate even at early to middle stages, and the choice of treatment strategy remains controversial [1]–[4]. AVC is the convergence of bilateral vocal cords and vestibular folds without a real boundary at the frontal larynx. The anterior aspect of AVC inserts into the thyroid cartilage with Broyle ligament. Since there is no perichondrium in the area of insertion, it is believed that rather than serving as a tumor barrier, the insertion area is actually a vulnerable area for invasion of AVC tumors into the thyroid cartilage. Glottic laryngeal tumors involving AVC may violate the contralateral larynx and thyroid cartilage by growing past this insertion area [13]. Given the fact that AVC is adjacent to multiple anatomical sub-regions of the larynx within a narrow space, it is not surprising that tumor cells can easily grow into the adjacent tissues. A tumor involving AVC may have already invaded the thyroid cartilage and surrounding sub-regions even it represents a small bump on the AVC surface. Therefore, the clinical stage of glottic laryngeal tumors involving AVC might be under-estimated, which may affect the choice of treatment strategy and prognosis [1], [14]. In line with this notion, we found that although all patients were graded as stage T1b stage before the procedure, four patients were actually detected with thyroid cartilage invasion by postoperative pathological evaluation and should be re-graded as T3 stage. This under-estimation in tumor staging may affect the prognosis significantly if appropriate treatment is not executed.

Open surgery is most frequently used to treat T1b squamous cell carcinoma of glottic larynx involving AVC. Since it can completely remove tumors along with the frontolateral thyroid cartilage, AVC and the corresponding vocal and vestibular folds, theoretically open surgery is suitable for AVC tumors at T1b stage, even some cases with thyroid cartilage involvement that should be graded as T3. However, specific limitations exist for present methods of open surgery. For example, laryngofissure is not sufficient in exposing lesions due to the involvement of bilateral vocal cords and AVC, which may lead to incomplete tumor removal and subsequent high recurrence. SCPL-CHEP involves removal of more normal tissue and leaves large surgical wounds, resulting in significant vocal dysfunction, poor postoperative voice quality and thus reduced life quality. The most commonly used procedure for tumors at T1b stage is AFVPL, in which the surgical defects are repaired by direct closure of laryngeal cavity, sternohyoid myofascia, sternohyoid perichondrium, platysma fascia, epiglottoplasty or modified Tucker's technique [10], [13], [15]. However, the integrity of thyroid cartilage framework is disrupted due to the removal of its frontolateral aspect along with the corresponding vocal cords and subglottic tissues. As a consequence, the postoperative vibration mode of vocal cords is severely impaired, leading to poor phonation [16]. Due to the narrow laryngeal cavity after reconstruction, friction damage occurs between the thyroid cartilage stump and repairing fascia or periosteum, causing the development of granuloma, scar hyperplasia and even laryngeal stenosis. In addition, epiglottoplasty results in lack of protection at laryngeal entrance, which may cause aspiration and feeding difficulties and thus reduced life quality [9], [17], [18].

In the MFHPL procedure we developed, the bilateral vocal cords where the tumors were located, and the middle aspect of thyroid cartilage corresponding to the AVC were completely removed under direct vision. The upper and lower cutting edges of stump larynx were sewed together to ensure the integrity of thyroid cartilage framework and the symmetry of glottis. More normal tissue was preserved to reduce the surgical wounds.

Our study demonstrated that prior to the surgery, there were no statistical differences in sex, age and differentiation degree of the tumors between MFHPL- and AFVPL-treated patients, suggesting that these two groups were comparable in general condition and extent of disease. No significant difference was detected in the incidence of postoperative complications of pneumonia and laryngeal fistula, recurrence rate, and survival rates between two groups. These results are consistent with those reported in both domestic and international studies [4], [19], supporting the feasibility of MFHPL procedure in treating stage T1b squamous cell carcinoma of glottic larynx involving AVC.

Of note, MFHPL showed a significant advantage over conventional AFVPL that were reflected by reduced incidence of postoperative laryngeal stenosis. This advantage may be attributed by multiple factors. First of all, the upper and lower parts of the thyroid cartilage and corresponding supra- and sub-glottis were preserved in order to maintain the integrity and symmetry of the laryngeal framework. Consequently, significant reduction in the space of laryngeal cavity was prevented post surgery. Secondarily, the glottis was reconstructed using the vestibular folds. The mucosal integrity was maintained in at least one side of the reconstructed AVC, which effectively resolved the postoperative issues of tissue adhesion and laryngeal web formation. In addition, the vestibular folds and cricothyroid membrane were sutured together during the larynx closure, minimizing the mucosal defects and thus reducing the incidence of laryngeal granuloma, laryngeal webs and laryngeal stenosis. As a result, the postoperative extubation rate reached 100% in the MFHPL group. Meanwhile, the MFHPL preserved the supra- and sub-glottic tissue, resulting in a reconstructed glottis with symmetry, smooth movement and high degree of closure. Consequently, the postoperative MPT was significantly longer in the MFHPL group than in the AFVPL group. Compared to the AFVPL procedure, the MFHPL procedure significantly attenuated the voice handicap in patients that was reflected by lower VHI score at all evaluation scales including functional, physiological, emotional and global assessment, indicating a better voice quality beneficial for daily verbal communication.

In conclusion, MFHPL procedure targeting stage T1b squamous cell carcinoma of glottis larynx involving AVC was demonstrated as an effective treatment with high voice quality preservation, low incidence of laryngeal stenosis and postoperative complications. Therefore, it has great potential in clinical application.
